# Surgical therapy of benign and low-grade malignant intramedullary chondroid lesions of the distal femur: intralesional resection and bone cement filling with or without osteosynthesis

**DOI:** 10.1007/s11751-018-0321-2

**Published:** 2018-11-03

**Authors:** Georg W. Omlor, Vera Lohnherr, Pit Hetto, Simone Gantz, Jörg Fellenberg, Christian Merle, Thorsten Guehring, Burkhard Lehner

**Affiliations:** 1 Center of Orthopaedics, Trauma Surgery and Paraplegiology, Heidelberg University Hospital, Schlierbacher Landstraße 200a, 69118 Heidelberg, Germany; 2 Department of Trauma and Orthopaedic Surgery, BG Trauma Center Ludwigshafen, University of Heidelberg, 67071 Ludwigshafen, Germany

**Keywords:** Chondroid lesion, Enchondroma, Atypical cartilaginous tumor, Chondrosarcoma, Femur, Compound osteosynthesis

## Abstract

Surgical treatment of benign and low-grade malignant intramedullary chondroid lesions at the distal femur is not well analyzed compared to higher-grade chondrosarcomas. Localization at the distal femur offers high biomechanical risks requiring sophisticated treatment strategy, but scientific guidelines are missing. We therefore wanted to analyze a series of equally treated patients with intralesional resection and bone cement filling with and without additional osteosynthesis. Twenty-two consecutive patients could be included with intralesional excision and filling with polymethylmethacrylate bone cement alone (*n* = 10) or with compound bone cement osteosynthesis using a locking compression plate (*n* = 12). Clinical and radiological outcome was retrospectively evaluated including tumor recurrences, complications, satisfaction, pain, and function. Mean follow-up was 55 months (range 7–159 months). Complication rate was generally high with lesion-associated fractures both in the osteosynthesis group (*n* = 2) and in the non-osteosynthesis group (*n* = 2). All fractures occurred in lesions that reached the diaphysis. No fractures were found in meta-epiphyseal lesions. No tumor recurrence was found until final follow-up. Clinical outcome was good to excellent for both groups, but patients with additional osteosynthesis had significantly longer surgery time, more blood loss, longer postoperative stay in the hospital, more complications, more pain, less satisfaction, and worse functional outcome. Intralesional resection strategy was oncologically safe without local recurrences but revealed high risk of biomechanical complications if the lesion reached the diaphysis with an equal fracture rate no matter whether osteosynthesis was used or not. Additional osteosynthesis significantly worsened final clinical outcome and had more overall complications. This study may help guide surgeons to avoid overtreatment with additional osteosynthesis after curettage and bone cement filling of intramedullary lesions of the distal femur. Meta-epiphyseal lesions will need additional osteosynthesis rarely, contrary to diaphyseal lesions with considerable cortical thinning.

## Introduction

Benign and low-grade malignant intramedullary chondroid tumors belong to the most common primary bone tumors mainly divided into enchondromas and atypical cartilaginous tumors (ACT/chondrosarcoma grade I according to older nomenclature) [1]. At the lower extremities, most large lesions are located at the femur [1–3]. Radiological and histological differences between aggressive large enchondromas and low-grade malignant ACT are hard to distinguish [4]. The risk of transformation of not only ACT but also large enchondromas into malignant higher-grade chondrosarcomas is evident [1, 5, 6]. Larger and clinically active lesions are at higher risk, but even small lesions should be routinely followed in the long term; however, there is little evidence about follow-up intervals and imaging modalities [4, 5, 7, 8]. Lesions which are large, painful, and radiologically aggressive with endosteal scalloping or soft tissue extension often receive surgery, to avoid further growth or transformation into higher-grade chondrosarcomas. Intralesional excision with vigorous curettage and additional filling with polymethylmethacrylate bone cement has been proven as the therapy of choice, whereas segmental resection with arthroplasty or other complex surgical reconstruction seems too aggressive with avoidable functional impairment [2, 5, 9–12]. Whereas metastases are rare [5, 13–17], local recurrences are frequent [2, 18–20] as well as instability of the femur and impairment of adjacent joint function [12, 21–23].

Sufficient long-term data on clinical outcome after surgically treated patients with intramedullary chondroid tumors at the distal femur is missing in the literature. Therefore, the aim of the present study was to retrospectively analyze our results after surgical therapy of these lesions. It is controversial whether additional osteosynthesis performed as a compound bone cement osteosynthesis with the intention to further stabilize the distal femur after intralesional excision, significantly influences long-term clinical results compared to bone cement filling alone [12, 21, 22]. We therefore analyzed the outcome after both strategies to compare recurrence rates, complications, and clinical function as well as patient satisfaction.

## Materials and methods

Between 2005 and 2017, we retrieved a total of 115 patients treated either conservatively or surgically for enchondroma or ACT of the distal femur at our university orthopedic oncology outpatient clinic. Systematic data collection was performed with our computerized tumor patients’ databank, and approval was given by the ethical committee of the Ruprecht-Karls-University of Heidelberg, Germany (vote number S-053/2017). Exclusion criteria were follow-up shorter than 6 months and syndromes with multiple lesions (morbus Ollier; Maffucci syndrome). Of the selected patients, only 26 underwent surgery because of clinical and radiological aggressiveness with larger size, endosteal scalloping, or soft tissue extension. Four surgically treated patients were excluded due to heterogeneous treatment including intralesional excision without further filling (*n* = 1), resection with cancellous bone filling with additional osteosynthesis (*n* = 1) and without (*n* = 2). Hence, 22 cases could be enrolled in the present series. All cases received intralesional excision via complete curettage of the lesion through a bone window of about 1 × 3 cm, additional use of a high-speed burr at the lesion margins and topical administration of H_2_O_2_ to kill potentially remaining tumor cells. The lesion was filled with polymethylmethacrylate bone cement to stabilize the bone and to minimize the risk for recurrence as polymerization heat of the exothermic reaction of the polymethylmethacrylate may kill potentially remaining tumor cells [12]. Compound bone cement osteosynthesis with a locking compression plate was performed in *n* = 12 cases due to expected higher instability, whereas *n* = 10 cases did not receive additional osteosynthesis as sufficient stability was expected with bone cement filling alone. Decision making was based on radiological features including lesion size, amount of scalloping, and localization as well as intra-operative presentation. Decision was made with the intention to assure sufficient stability and avoid postoperative fracture but also choosing the least invasive treatment as possible. Adequate exposure was mandatory to verify complete intralesional excision. Both groups did not show significant differences in their medical records considering preoperative pain, range of motion, weight, age, gender, and comorbidities. All patients underwent an equal postoperative after-treatment with physical therapy for mobilization. Weight bearing of the operated leg was limited to 20 kg for 4–6 weeks before full weight bearing was recommended.

Full radiological evaluation with X-rays and MRIs was performed initially and regularly with intervals of initially 6 and later 12 months until final follow-up. In limited cases, CT scans and additional scintigraphy were also performed. Imaging was evaluated by musculoskeletal radiologists subspecialized in bone and soft tissue tumor diagnostics.

For clinical evaluation, physical examination was assessed in our orthopedic oncology outpatient clinic and documented in the tumor patients’ databank. Full patient demographics and clinical histories were documented with detailed information on surgical treatment, histological analysis, potential recurrences, and complications. At final follow-up, pain, patient satisfaction, and functional outcome were additionally evaluated with a questionnaire-based telephone interview. Visual analog scale ratings for pain and patient satisfaction from 0 to 10 were evaluated. Limitations and musculoskeletal function of the adjacent knee joint were semiquantitatively evaluated by the oxford knee score with ratings from 0 to 48 [24]. Ratings from 40 to 48 indicate good-to-satisfactory joint function, 30–39 mild-to-moderate limitations, 20–29 moderate limitations, and 0–19 severe limitations.

For statistical analysis, descriptive statistics were presented as mean and range for numerical variables and for frequencies with corresponding percentages for categorical variables. To compare the differences, Student t tests, Mann–Whitney U tests, and Kruskal–Wallis tests were performed depending on scale level and distribution of the data. Statistical significance was assumed at a *p* value < 0.05.

## Results

Mean follow-up was 55 months (range 7–159 months). Patient demographics and lesion characteristics and surgical parameter are presented in Table 1. Additional osteosynthesis caused significantly longer surgery time, more blood loss, and longer postoperative stay in the hospital. None of the cases revealed tumor recurrence, no matter whether additional osteosynthesis was used or not.

**Table 1:** Patient demographics, surgical parameter, and characteristics of the lesions

	Resection + bone cement (*n* = 10)	Resection + bone cement + osteosynthesis (*n* = 12)	*p* value
Gender			0.79
Male	*n* = 2	*n* = 3	
Female	*n* = 8	*n* = 9	
Age median (range)	51 (32–77) years	49 (37–61) years	0.64
Histology			0.45
Enchondroma	*n* = 8	*n* = 8	
ACT	*n* = 2	*n* = 4	
Initial tumor size mean (standard deviation)	4.8 (1.5) cm	6.3 (2.8) cm	0.13
Enchondroma	4.4 (1.2) cm	5.7 (2.7) cm	0.24
ACT	6.3 (2.5) cm	7.5 (3.0) cm	0.64
Recurrence	0	0	
Surgery time median (range)	62 (42–78) min	136 (99–201) min	< 0.0001
Length of stay median (range)	6 (3–9) days	9 (6–16) days	0.006
Blood loss median (range)	125 (50–1000) ml	402 (100–800) ml	0.001
Complications			0.45
Postoperative fracture	2	2	
Intra-articular screw		1	
Plate irritation with need for plate removal		1	

### Radiological outcome

**Fig. 1:**
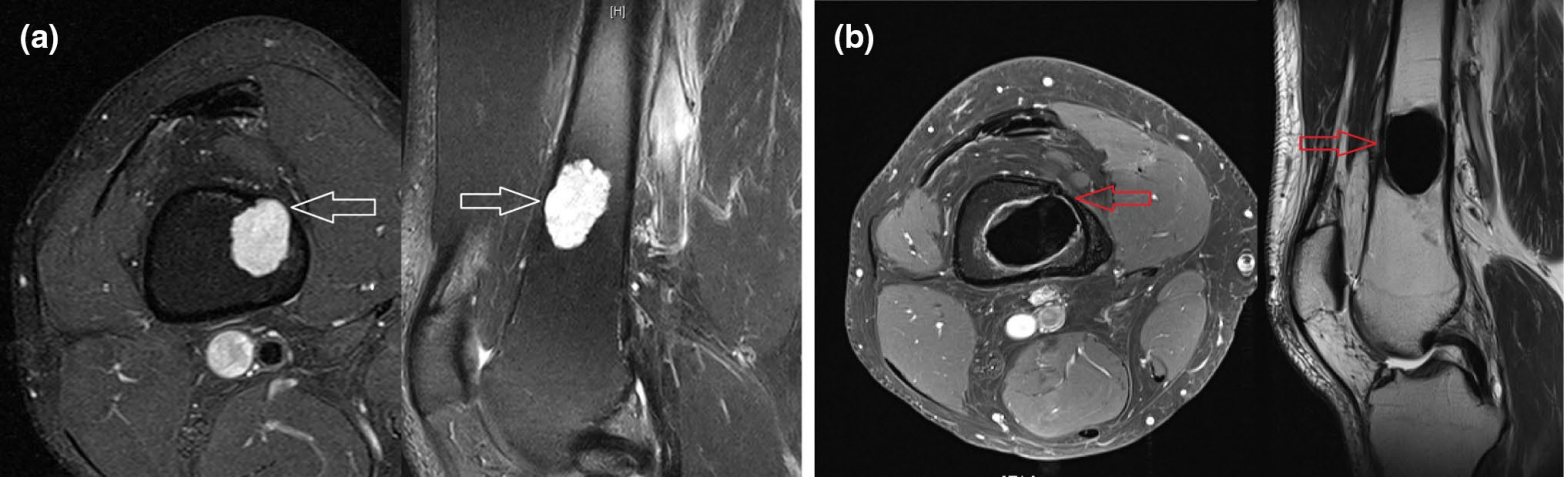
Preoperative MRI with endosteal scalloping and soft tissue extension (white arrows) of the lesion located in the metaphysis and diaphysis (**a**). Treatment was performed with intralesional excision and bone cement filling without additional osteosynthesis (**b**). Eighteen months postoperatively cortical bone grew over the initial scalloping zone and bone window (red arrows) resulting in a stable distal femur. The bone cement filling is regularly surrounded by edema (white margin around the filling), which must be distinguished from local recurrence (color figure online)

**Fig. 2:**
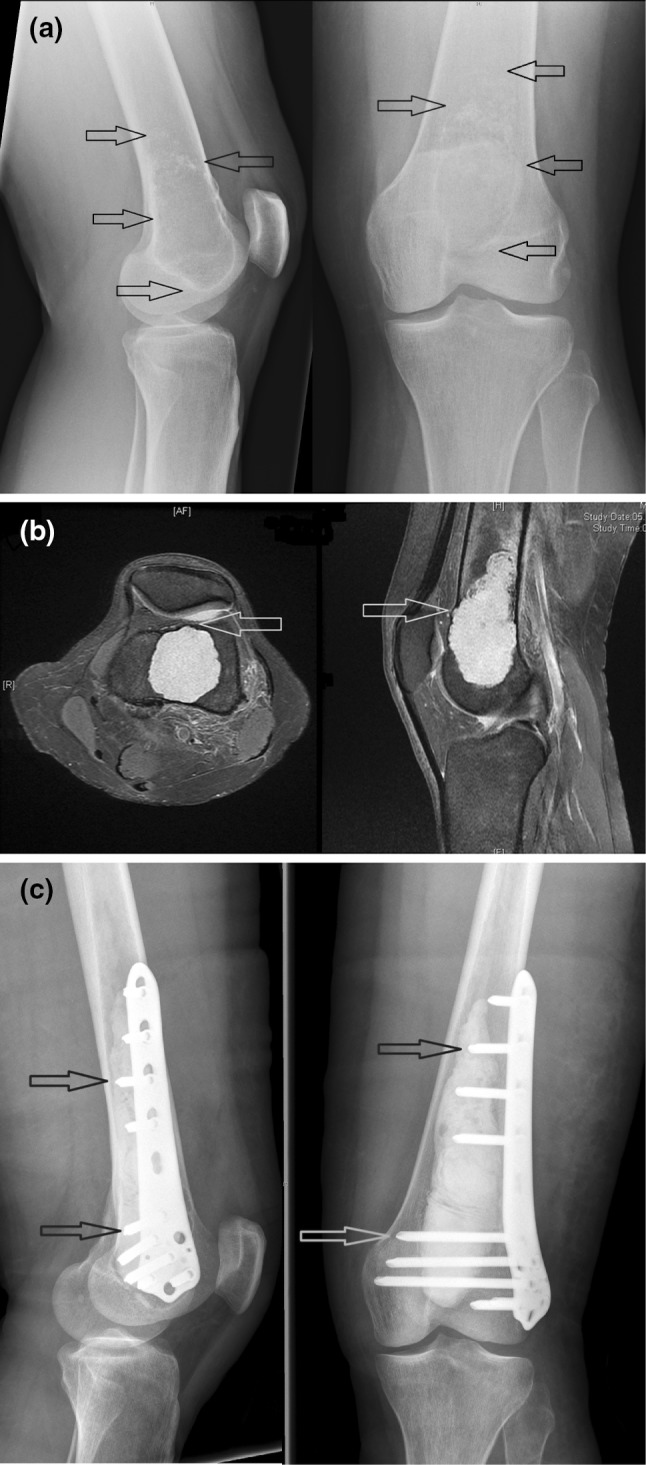
Preoperative X-ray (**a**) depicts a large atypical cartilaginous tumor (ACT) (black arrows) with typical intralesional popcorn-like ossification in the proximal part (red arrows). Preoperative MRI (**b**) shows endosteal scalloping and soft tissue extension (red arrows). Postoperative X-ray (**c**) documents the compound bone cement plate osteosynthesis with integration of the screws of the locking compression plate into the polymethylmethacrylate bone cement (red arrows) to increase stability after intralesional excision of the ACT (color figure online)

Initial imaging did not show significant differences in size (mean 4.8 cm versus 6.3 cm; *p* = 0.13) or other lesion characteristics as endosteal scalloping or soft tissue extension. All 22 lesions involved the metaphysis of the distal femur. Ten out of 12 lesions from the osteosynthesis group reached into the diaphysis compared to 5 out of 10 lesions from the non-osteosynthesis group. At final follow-up, imaging revealed sufficient stability in all cases. In those cases treated without additional osteosynthesis, cortical bone overgrew the initially open bone window at the entry site of the excision (Fig. 1). In case of additional osteosynthesis, locking compression plates were integrated into the bone cement as a compound osteosynthesis (Fig. 2). Endoprothetic reconstruction of the knee joint was not needed and not performed in the patient series during the analyzed interval.

### Clinical outcome

Overall clinical outcome was good to excellent. Patients treated with additional osteosynthesis, however, presented significantly worse values for overall satisfaction with the treatment, overall pain, and function. Detailed results and p values are shown in Table 2.

**Table 2:** Clinical outcome after intralesional excision and bone cement filling depending on whether additional osteosynthesis was performed or not

	Resection + bone cement	Resection + bone cement + osteosynthesis	*p* value
Satisfaction from 0 to 10 mean (standard deviation)	9.88 (0.35)	7.43 (1.81)	0.003
Pain from 0 to 10 mean (standard deviation)	0.3 (0.7)	3.0 (1.2)	0.001
function from 0 to 48 in the Oxford Knee Score mean (standard deviation)	47.0 (2.1)	36.6 (8.8)	0.002

### Complications

**Fig. 3:**
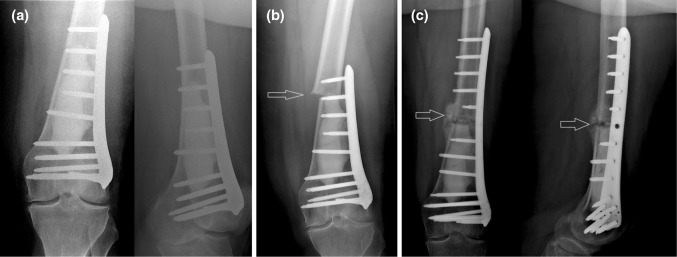
X-ray immediately after intralesional excision and osteosynthesis (**a**) followed by early fracture at the proximal part (red arrows) of the bone cement (**b**). Revision surgery with re-osteosynthesis with a longer locking compression plate resulted in callus bone healing (red arrows) 3 months after fracture (**c**) (color figure online)

Complication rate was generally high with six complications in 22 cases, and it was almost two times higher in cases with additional osteosynthesis (Table 1). Four of those 12 patients who received bone cement filling and additional osteosynthesis with a locking compression plate showed postoperative complications. One patient complained about pain which was attributed to an irritating locking screw. The screw was removed and replaced by a shorter one. Two patients had peri-implant fractures and received re-osteosynthesis with longer locking compression plates. Of those, one patient had an early dislocated postoperative fracture 14 days after surgery without obvious trauma while getting out of the car (Fig. 3). The second patient developed a non-dislocated fracture at the proximal border of the bone cement filling after an adequate trauma with a fall at home 3 months after surgery. Another patient suffered from plate irritation at the soft tissues next to the distal lateral femur requiring revision surgery with plate removal.

**Fig. 4:**
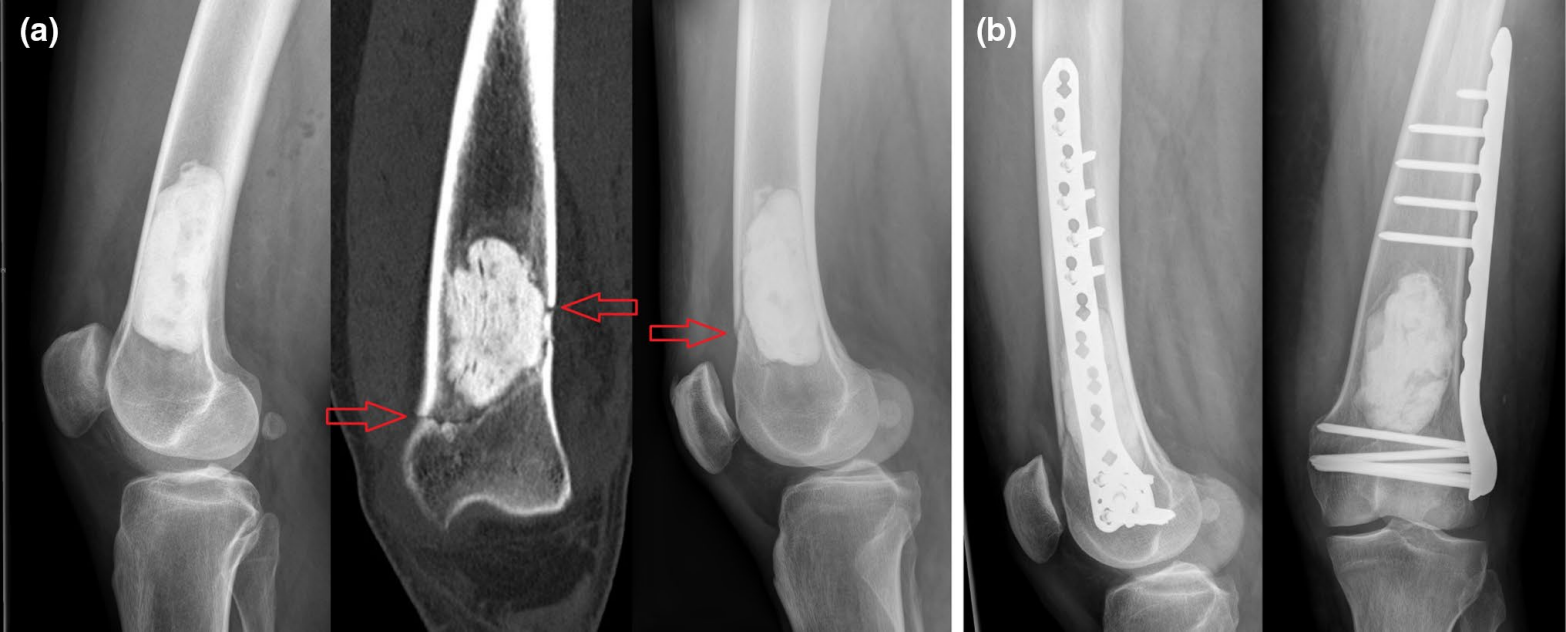
Postoperative X-ray after intralesional excision and filling with polymethylmethacrylate bone cement without additional osteosynthesis (**a**). Three months after surgery, a fracture was caused by an adequate sport trauma. The non-dislocated fracture is visible in CT and lateral X-ray (red arrows). Revision surgery was performed with osteosynthesis using a large locking compression plate without revision of the bone cement filling (**b**) (color figure online)

Two of those 10 patients that underwent intralesional resection with bone cement filling without osteosynthesis had postoperative fractures. One patient had a non-dislocated fracture beneath the cortical bone window early after 6 weeks while standing on one leg after mobilization with full weight bearing. The other patient had a metaphyseal fracture at the distal border of the bone cement filling 3 months after surgery after an adequate sport trauma with a fall (Fig. 4). Fractures were treated with locking compression plates without revision of the initial bone cement filling. No further fractures occurred until final follow-up.

## Discussion

We retrospectively analyzed a series of 22 surgically treated patients with enchondroma and ACT at the distal femur adjacent to the knee joint. One group was treated only with intralesional tumor resection and bone cement filling (*n* = 10), whereas the other group underwent the same procedure with additional use of a locking compression plate (*n* = 12) as a compound bone cement osteosynthesis. Data on surgically treated patients with such benign to low-malignant cartilaginous bone tumors at the distal femur is rare and larger series are not available [12]. This mono-center study could analyze 22 otherwise equally treated patients with two different surgical strategies at the distal femur with a mean follow-up of 55 months. This study may help guide surgeons to avoid overtreatment as we could prove significant clinical disadvantages after additional plate osteosynthesis compared to bone cement filling alone.

Until final follow-up, no tumor recurrence was found, so intralesional tumor resection strategy with rigorous curettage and use of polymethylmethacrylate bone cement was successful.

There are no scientific guidelines on how to make the decision whether additional osteosynthesis should be used or not. For the presented series, decision was made based on preoperative imaging and intra-operative presentation to achieve sufficient stability. Re-analysis of preoperative imaging could not verify objective differences regarding cortical destruction, scalloping, and lesion size between both groups. Comparability of osteosynthesis and non-osteosynthesis cases, however, was limited by the retrospective study design and the small group size with a potential selection bias as more complicated lesions might have been selected for osteosynthesis.

Lesions which were treated without osteosynthesis were more often located in the metaphysis closer to the epiphysis than lesions receiving additional osteosynthesis. Metaphyseal lesions that are located closer to the epiphysis and more distant from the diaphysis may have a lower risk for fracture due to lower biomechanical forces [12]. This has not been proven in the literature so far, but in the present study, none of the metaphyseal lesions that did not reach the diaphysis had fractures whereas all fractures occurred in lesions reaching into the diaphysis. Hence, surgery without additional osteosynthesis might be preferred in lesions that do not reach the diaphysis of the distal femur. Statistics with further subgroups (closer to the epiphysis versus closer to the diaphysis) were not presented in the results section, due to small group sizes and hence limited power. Re-analysis of statistics after exclusion of those lesions closer to the epiphysis suggested equal outcome with similar lesion sizes and still superior clinical results in the non-osteosynthesis group with still significantly higher satisfaction, lower pain, and better function.

Surgeries were only performed by a small group of subspecialized surgeons from the orthopedic tumor department, so significant bias due to different surgeons is not likely for the presented series. All surgeons used both strategies—with and without additional osteosynthesis—without significant differences in frequency.

Theoretically, additional osteosynthesis offers the advantage of higher stability immediately after surgery with few restrictions for postoperative mobilization and weight bearing and reduced risk of fracture [25]. Nevertheless, we found two postoperative fractures in both groups with and without osteosynthesis, so we could not prove better prevention of fractures in case of additional osteosynthesis. All fractures happened in lesions closer to the diaphysis of the distal femur, whereas lesions closer to the epiphysis did not show fractures, so these lesions might be more stable with less need for additional osteosynthesis. Other complications such as intra-articular screw position and plate irritation of soft tissues with need for revision surgery were a clear disadvantage of osteosynthesis compared to bone cement filling without additional implant [26].

Heterogeneous local recurrence rates have been reported in the literature for both aggressive enchondromas and ACT [2, 18–20]. Our follow-up data without recurrences in all 22 cases supports the success of our intralesional resection strategy. Most enchondromas do not need surgery but especially large and active ones should be radiologically followed, whereas ACT is more often treated surgically with the problem of difficult radiologic and histologic distinguishability from enchondroma [4, 7, 13]. Intralesional resection with curettage [2, 5, 9–11, 17, 20, 23, 27] instead of more invasive wide resection [10, 28] is accepted as the standard surgical strategy for these rather benign to only low-malignant cartilaginous bone tumors [29]. Additional use of a high-speed burr may eliminate remaining tumor cells at the margins of the lesion by heat destruction as well as the exothermic reaction during bone cement polymerization [27, 30]. We did not use further cryotherapy or phenol but H_2_O_2_ with similar effects to decrease recurrence rates [23].

Recurrence rate might be related to the surgical strategy, as well. If additional osteosynthesis is not planned, the surgeon might be more concerned about bone stability after intralesional resection and hence exposure and resection of the tumor might be less radical and less comprehensive resulting in—at least theoretically—higher risk for tumor recurrence. Our data do not support this, as we did not find differences in the aggressiveness of the curettage nor in recurrences in both groups.

The clinical outcome was overall good to excellent. Even the worst results of both groups were related to a satisfactory yield. Although the presented series is small due to the rare indication for the presented surgeries, the analyzed parameter already revealed statistically significant differences between both groups. Additional osteosynthesis caused significantly more pain, less function, and less satisfaction with the treatment and was associated with significantly longer surgery time and more blood loss.

Sufficient data are missing in the literature whether additional osteosynthesis is beneficial or not. Perisano et al. suggested additional osteosynthesis after intralesional excision for all lesions that were larger than 5 cm. Osteosynthesis was also suggested for all lesions of the distal femur irrespective of their size due to higher risk of fractures at this location [12]. Our results confirm the high risk of early fracture at the distal femur but also show distinct advantages if additional osteosynthesis was avoided. Patients were more satisfied and had less pain and better function as well as less complication if osteosynthesis was not used. At the long term, sufficient stability was restored as cortical bone could overgrow the cement filling at the bone window at the entry site. To facilitate this, we recommend a bone cement technique which only fills the inner lesion without overlapping the cortical wall of the femur. In the present series, the risk for fracture was only evident early after surgery as all fractures occurred within 3 months, which may suggest a more cautious postoperative regime without sport activities for up to 3 or even 6 months. To minimize soft tissue trauma in case of osteosynthesis and as the implants were recommended not to be removed later on, we rather used short plates based on the concept of a compound bone cement osteosynthesis with integration of the screws in the bone cement, where sufficient stability is expected without longer fixation above the lesion. The use of longer plates, however, would have presumably reduced the fracture rate in the osteosynthesis group. To obtain objective data, we encourage further biomechanical cadaver studies on this topic, to validate the stability after intralesional bone cement filling without osteosynthesis and with compound osteosynthesis using different sized locking compression plates. Furthermore, the results from the present study support the initiation of multicenter studies on this issue, to reduce bias and increase statistical power by obtaining larger series, which may define further criteria for decision making.

In conclusion, the present series suggests that intralesional excision and bone cement filling without additional osteosynthesis may be favorable even at the biomechanically heavily loaded distal femur due to significantly better clinical results, less surgery time, and less complications. High risk of early postoperative fracture should be considered for both treatment options. Despite the clinical disadvantages we, however, still recommend additional osteosynthesis for all cases with diaphyseal location and considerable thinning out of the cortical wall. Osteosynthesis should also not be avoided, if curettage would otherwise be limited or performed less aggressive. Lesions located closer to the epiphysis seem less at risk for fracture, which should be considered for decision making to avoid overtreatment.

## References

[1] Jo VY, Fletcher CD (2014). WHO classification of soft tissue tumours: an update based on the 2013 (4th) edition. Pathology.

[2] Brown MT, Gikas PD, Bhamra JS, Skinner JA, Aston WJ, Pollock RC, Saifuddin A, Briggs TW (2014). How safe is curettage of low-grade cartilaginous neoplasms diagnosed by imaging with or without pre-operative needle biopsy?. Bone Joint J.

[3] Donthineni R, Ofluoglu O (2010). Solitary enchondromas of long bones: pattern of referral and outcome. Acta Orthop Traumatol Turc.

[4] Deckers C, Schreuder BH, Hannink G, de Rooy JW, van der Geest IC (2016). Radiologic follow-up of untreated enchondroma and atypical cartilaginous tumors in the long bones. J Surg Oncol.

[5] Andreou D, Gilg MM, Gosheger G, Werner M, Hardes J, Pink D, Leithner A, Tunn PU, Streitbürger A (2016). Metastatic potential of grade I chondrosarcoma of bone: results of a multi-institutional study. Ann Surg Oncol.

[6] Altay M, Bayrakci K, Yildiz Y, Erekul S, Saglik Y (2007). Secondary chondrosarcoma in cartilage bone tumors: report of 32 patients. J Orthop Sci.

[7] Sampath Kumar V, Tyrrell PN, Singh J, Gregory J, Cribb GL, Cool P (2016). Surveillance of intramedullary cartilage tumours in long bones. Bone Joint J.

[8] Crim J, Schmidt R, Layfield L, Hanrahan C, Manaster BJ (2015). Can imaging criteria distinguish enchondroma from grade 1 chondrosarcoma?. Eur J Radiol.

[9] Van der Geest IC, de Valk MH, de Rooy JW, Pruszczynski M, Veth RP, Schreuder HW (2008). Oncological and functional results of cryosurgical therapy of enchondromas and chondrosarcomas grade 1. J Surg Oncol.

[10] Hickey M, Farrokhyar F, Deheshi B, Turcotte R, Ghert M (2011). A systematic review and meta-analysis of intralesional versus wide resection for intramedullary grade I chondrosarcoma of the extremities. Ann Surg Oncol.

[11] Hanna SA, Whittingham-Jones P, Sewell MD, Pollock RC, Skinner JA, Saifuddin A, Flanagan A, Cannon SR, Briggs TW (2009). Outcome of intralesional curettage for low-grade chondrosarcoma of long bones. Eur J Surg Oncol.

[12] Perisano C, Barone C, Stomeo D, Di Giacomo G, Vasso M, Schiavone Panni A, Maccauro G (2016). Indications for prophylactic osteosynthesis associated with curettage in benign and low-grade malignant primitive bone tumors of the distal femur in adult patients: a case series. J Orthop Traumatol.

[13] Evans HL, Ayala AG, Romsdahl MM (1977). Prognostic factors in chondrosarcoma of bone: a clinicopathologic analysis with emphasis on histologic grading. Cancer.

[14] Andreou D, Ruppin S, Fehlberg S, Pink D, Werner M, Tunn PU (2011). Survival and prognostic factors in chondrosarcoma: results in 115 patients with long-term follow-up. Acta Orthop.

[15] Streitbürger A, Ahrens H, Balke M, Buerger H, Winkelmann W, Gosheger G, Hardes J (2009). Grade I chondrosarcoma of bone: the Münster experience. J Cancer Res Clin Oncol.

[16] Funovics PT, Panotopoulos J, Sabeti-Aschraf M, Abdolvahab F, Funovics JM, Lang S, Kotz RI, Dominkus M (2011). Low-grade chondrosarcoma of bone: experiences from the Vienna Bone and Soft Tissue Tumour Registry. Int Orthop.

[17] Campanacci DA, Scoccianti G, Franchi A, Roselli G, Beltrami G, Ippolito M, Caff G, Frenos F, Capanna R (2013). Surgical treatment of central grade 1 chondrosarcoma of the appendicular skeleton. J Orthop Traumatol.

[18] Errani C, Tsukamoto S, Ciani G, Akahane M, Cevolani L, Tanzi P, Kido A, Honoki K, Tanaka Y, Donati DM (2017). Risk factors for local recurrence from atypical cartilaginous tumour and enchondroma of the long bones. Eur J Orthop Surg Traumatol.

[19] Schwab JH, Wenger D, Unni K, Sim FH (2007). Does local recurrence impact survival in low-grade chondrosarcoma of the long bones?. Clin Orthop Relat Res.

[20] Donati D, Colangeli S, Colangeli M, Di Bella C, Bertoni F (2010). Surgical treatment of grade I central chondrosarcoma. Clin Orthop Relat Res.

[21] Hirn M, de Silva U, Sidharthan S, Grimer RJ, Abudu A, Tillman RM, Carter SR (2009). Bone defects following curettage do not necessarily need augmentation. Acta Orthop.

[22] Kundu ZS, Gupta V, Sangwan SS, Rana P (2013). Curettage of benign bone tumors and tumor like lesions: a retrospective analysis. Indian J Orthop.

[23] Dierselhuis EF, Gerbers JG, Ploegmakers JJ, Stevens M, Suurmeijer AJ, Jutte PC (2016). Local treatment with adjuvant therapy for central atypical cartilaginous tumors in the long bones: analysis of outcome and complications in one hundred and eight patients with a minimum follow-up of two years. J Bone Joint Surg Am.

[24] Collins NJ, Misra D, Felson DT, Crossley KM, Roos EM (2011). Measures of knee function: International Knee Documentation Committee (IKDC) Subjective Knee Evaluation Form, Knee Injury and Osteoarthritis Outcome Score (KOOS), Knee Injury and Osteoarthritis Outcome Score Physical Function Short Form (KOOS-PS), Knee Outcome Survey Activities of Daily Living Scale (KOS-ADL), Lysholm Knee Scoring Scale, Oxford Knee Score (OKS), Western Ontario and McMaster Universities Osteoarthritis Index (WOMAC), Activity Rating Scale (ARS), and Tegner Activity Score (TAS). Arthritis Care Res (Hoboken).

[25] Dijstra S, Wiggers T, van Geel BN, Boxma H (1994). Impending and actual pathological fractures in patients with bone metastases of the long bones. A retrospective study of 233 surgically treated fractures. Eur J Surg.

[26] Busam ML, Esther RJ, Obremskey WT (2006). Hardware removal: indications and expectations. J Am Acad Orthop Surg.

[27] Di Giorgio L, Touloupakis G, Vitullo F, Sodano L, Mastantuono M, Villani C (2011). Intralesional curettage, with phenol and cement as adjuvants, for low-grade intramedullary chondrosarcoma of the long bones. Acta Orthop Belg.

[28] Staats K, Panotopoulos J, Tiefenboeck TM, Windhager R, Funovics PT (2017). Computer navigation-assisted surgery for musculoskeletal tumors: a closer look into the learning curve. Eur J Orthop Surg Traumatol.

[29] Chen X, Yu LJ, Peng HM, Jiang C, Ye CH, Zhu SB, Qian WW (2017). Is intralesional resection suitable for central grade 1 chondrosarcoma: a systematic review and updated meta-analysis. Eur J Surg Oncol.

[30] Piccioli A, Ventura A, Maccauro G, Spinelli MS, Del Bravo V, Rosa MA (2011). Local adjuvants in surgical management of bone metastases. Int J Immunopathol Pharmacol.

